# Managing Aesthetic Needs in Prescription Medication‐Driven Rapid Weight Loss Patients: Results of an International Consensus. The Clinician Perspective

**DOI:** 10.1111/jocd.70644

**Published:** 2026-01-20

**Authors:** Andreas Nikolis, Michael T. Somenek, Steven Dayan, Hugues Cartier, Sabrina G. Fabi, Luiz Avelar, Johnny Franco, Konstantin Frank, Alessandra Haddad, Mohammed A. Alsufyani, Jeff Huang, Inna Prygova, Tyler Safran

**Affiliations:** ^1^ Clinical Research Unit Erevna Innovations Inc Westmount Quebec Canada; ^2^ Division of Plastic and Reconstructive Surgery McGill University Montréal Quebec Canada; ^3^ Somenek+Pittman MD Advanced Plastic Surgery Washington DC USA; ^4^ Department of Otolaryngology University of Illinois Chicago Illinois USA; ^5^ Saint‐Jean Dermatology Center Arras France; ^6^ Cosmetic Laser Dermatology, San Diego University of California‐San Diego San Diego California USA; ^7^ DOMANI Clinic Belo Horizonte Brazil; ^8^ Austin Plastic Surgeon Austin Texas USA; ^9^ Department of Plastic, Hand and Reconstructive Surgery University Hospital Regensburg Regensburg Germany; ^10^ Department of Plastic Surgery Federal University of São Paulo UNIFESP Sao Paulo Brazil; ^11^ AHaddad Medicine and Surgery Clinic Sao Paulo Brazil; ^12^ Department of Dermatology Prince Sultan Medical Military City Riyadh Saudi Arabia; ^13^ L'excellence Clinica Taipei Taiwan; ^14^ Galderma Uppsala Sweden

**Keywords:** consensus, GLP1, prescription medication, weight loss

## Abstract

**Introduction:**

The global prevalence of obesity has continued to increase at alarming rates. More recently, there has been an exponential increase in the usage of GLP1 agonists in both clinically obese patients and those seeking weight loss. As a result, there has been an influx of patients who are noticing substantial weight loss and changes to their bodies. Given this surge, it is of utmost importance to have homogeneity in clinical guidelines. A consensus panel was created to discuss clinical scenarios, definitions, and set the stage for the appropriate treatment of patients who have undergone massive weight loss.

**Methods:**

To set standards for nonsurgical rejuvenation of patients at different ages, weight loss patterns, and genders, a consensus panel of 10 panelists was created with advanced expertise and knowledge on this patient population. Each panelist filled out a survey that addressed panelist demographics, including practice patterns and patient demographics, as well as treatment guidelines in both male and female patients. Following the survey, the panelists met to discuss preliminary results and obtain qualitative data to support their conclusions on the criteria.

**Results:**

All 10 respondents (100%) were able to complete the survey in its entirety. The panel demonstrates the need to assess and address each treatment region individually in these patients. It also emphasizes the need to consider the appropriate range of volumization based on factors such as age, gender, and the degree of weight loss. While the consensus suggests initiating biostimulator treatment concurrently with weight loss to mitigate fat pad deflation and skin laxity, uncertainties remain regarding the optimal timing and dosing.

**Conclusion:**

In conclusion, this consensus panel represents the first international effort to provide clinical guidance specifically for patients experiencing medication‐derived weight loss (MDWL). When creating a treatment plan for the MDWL patient, following them throughout the weight loss journey is essential to understand the more global volume changes that they may be observing.

## 1 Introduction

The global prevalence of obesity has continued to increase at alarming rates [1]. More recently, there has been an exponential increase in the usage of GLP1 agonists in both clinically obese patients and those seeking weight loss [2]. As a result, there has been an influx of patients who are noticing substantial weight loss and changes to their bodies. This comes at a time when social media–fueled interest in beauty standards has peaked, and patients' interest in obtaining treatments. In terms of bodily changes to weight fluctuations, patients are noticing the obvious changes in truncal and abdominal girth, but they are also noticing substantial changes in facial aging [3]. Many of the brand‐name GLP1 agonists have taken the spotlight, leading to their association with sagginess, loss of skin luster, fat pad atrophy, and a gaunt appearance [3].

Given this surge, it is of utmost importance to have homogeneity in clinical guidelines. A consensus panel was created to discuss clinical scenarios, definitions, and set the stage for the appropriate treatment of patients who have undergone massive weight loss [4]. This primary consensus panel agreed on using the term Medication‐derived weight loss (MDWL) to avoid brand names of GLP‐1 agonists, as well as to help differentiate the different mechanisms for weight loss (gradual, bariatric) [4]. Respondents agreed that MDWL treatments should be stratified for gender (60.0%), as well as age (100%) [4]. Seventy percent (70%) found that MDWL had a significant impact on the skin, and as a result, consensus agreed that treatment with biostimulators should begin concurrently with weight loss [4]. One of the consensus discussion topics was the importance of utilizing the entire breadth of the physician's armamentarium in both treatment and prevention [4]. Biostimulators have been shown to be an excellent adjunct to facial rejuvenation, as well as on their own have demonstrated favorable results in improving the severity of moderate/severe cheek lines and wrinkles, all while improving skin quality [5, 6]. Coupled with hyaluronic acid fillers, these treatment tools provide aesthetic providers with tools to help combat features of MDWL facial changes. Of note, each of the GLP1 agonists exhibits different effects on the patient, but this is beyond the scope of this article.

There is much variability in terms of anatomic regions to treat and volumes to utilize. Therefore, the goal of this current consensus is to set standards for nonsurgical rejuvenation of patients at different ages, weight loss patterns, and genders.

## 2 Methods

### 2.1 Steering Committee

A steering committee was created to help oversee the survey, as described previously [4]. The committee consisted of key stakeholders (representatives from endorsing organizations), senior leadership, as well as project managers with expertise in consensus methodology. Together and utilizing results from the prior consensus panel, they identified the key classification of grouping (age, gender, BMI loss, as well as speed of weight loss). For this survey, rapid weight loss is defined as weight loss in less than 5 weeks. Gradual weight loss is defined as weight loss in > 15 weeks. Groups were also split between > 20% BMI lost and < 20% BMI lost.

Additionally, they identified possible panelists with advanced expertise and knowledge on this patient population. The possible panelists were selected by said steering committee, avoiding bias. Panelists (*n* = 10) were invited, and all 10 had accepted. Geographic location was selected to best represent not only areas where weight loss medications are accessible and in high demand, but also to better represent a range of ethnicities and aesthetic goals.

### 2.2 Survey Design

After discussion with the steering committee and a review of the available evidence, an initial online survey was distributed to the panelists. This survey addressed panelist demographics, including practice patterns and patient demographics, which were previously published [4]. This clinical portion was created to address treatment guidelines in both male and female patients. Panelists were asked to address which anatomic regions they would treat with both biostimulators and fillers in patients at different weight loss ranges, ages, as well as rapidity of weight loss.

Based on the steering committee's findings, the three age groups were determined to be 20–40 years old, 41–60 years old, and 61+. Additionally, as mentioned above, weight loss was categorized as less than or greater than 20% of BMI lost.

**FIGURE 1:**
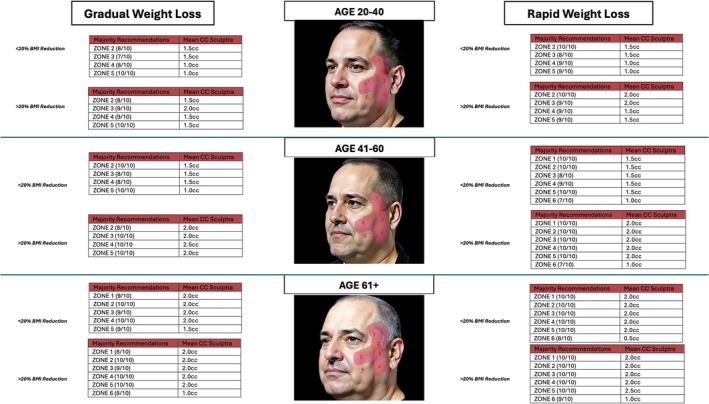
Male biostimulator.

Anatomic regions of treatment were created for each gender and age range. They were labeled Zones 1–6. This was created based on the steering committee and key opinion leader discussions (Figure 1). Additionally, volumes required for poly‐l‐lactic acid injections were based on standard reconstitution of 8 cc sterile water and 1 cc lidocaine without epinephrine (9 cc/syringe). To simplify volumes, consensus volumes were rounded to the nearest 0.5 cc (2.4 cc of PLLA > 2.5 cc in consensus).

### 2.3 Postsurvey Discussion

Following the survey, the panelists met to discuss preliminary results and obtain qualitative data to support their conclusions on the criteria. These qualitative discussions helped to shape the discussion of this article and to better understand the anatomic changes experienced in the MDWL patient.

### 2.4 Statistical Analysis

Following the survey results, statistical analysis was performed for each group. Consensus agreement was determined to be 70% or more, based on the prior consensus document. To understand how certain elements affect volume increases, mean percentage differences were calculated. Additionally, mean values were calculated for each treatment area. Statistical analysis was performed using SPSS (IBM, United States) and *p* values < 0.05 were considered significant.

## 3 Results

**FIGURE 2:**
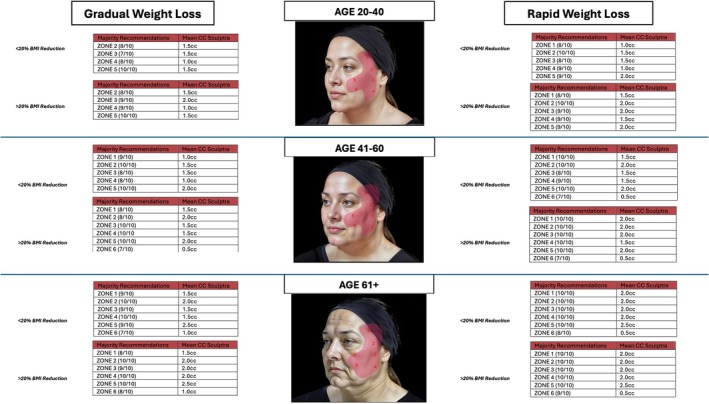
Female biostimulator.

**FIGURE 3:**
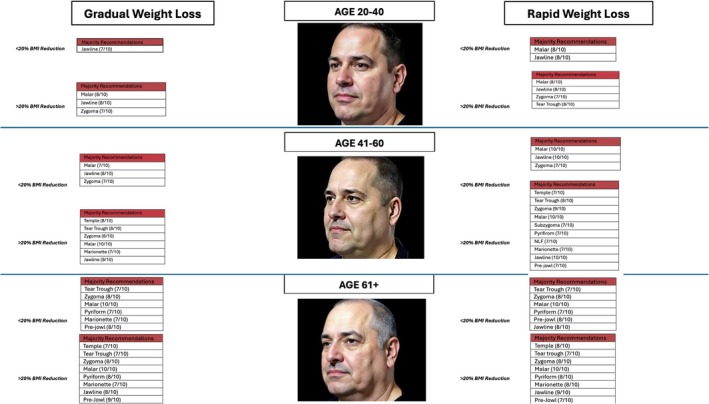
Male filler.

**FIGURE 4:**
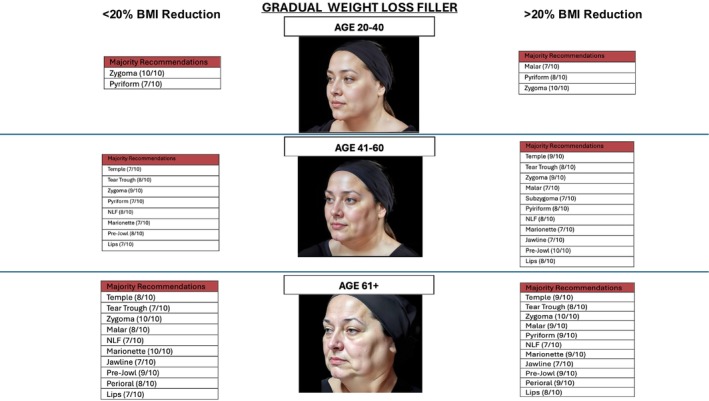
Female filler gradual weight loss.

**FIGURE 5:**
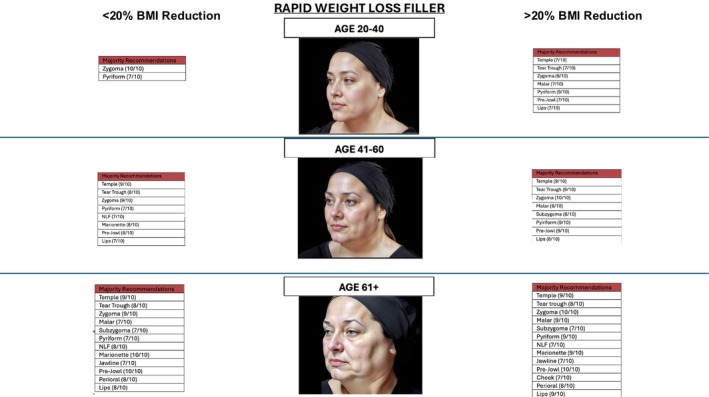
Female filler rapid weight loss.

All 10 respondents (100%) were able to complete the survey in its entirety (Figures 1 and 2), demonstrate zone division, as well as the priority of the treatment algorithm. Diagrammatic representation of both male and female treatment algorithms can be found in (Figures 1, 2, 3, 4, 5).

### 3.1 Females—Biostimulators

In terms of consensus, in the ages 20–40 groups, gradual weight loss in both < 20% and > 20% BMI, Zones 2,3,4, and 5 all achieved consensus. Interestingly, in the rapid groups, Zone 1 additionally achieved consensus in both < 20% and > 20% BMI. Zone one was added to take advantage of the templar lifting effect [7]. As age increased, consensus was achieved for all groups. Only in the 40‐ to 60 year‐old, < 20% BMI, and gradual weight loss Zone 6 was not added. Zone 6, which corresponds to prejowl, would need to be blended and strengthened in older patients and with more rapid, larger weight loss.

In terms of the volume of biostimulator, the average volume injected per zone was calculated via the survey. To obtain total volumes, the mean cc of injection per zone that achieved consensus was added. The total volumes indicated are per side of the face. The total volume for ages 20–40 BMI < 20% gradual is 5.5 cc (4 zones), for rapid is 7.0 cc (5 zones). In the same age group, for > 20% BMI gradual reduction, the injection volume is 6.0 cc (4 zones), and for rapid is 9.0 cc (5 zones). In the 40‐ 60‐year‐old cohort, in the BMI < 20% reduction gradually, the volume injected is 7.0 cc (5 zones). In the rapid group, the volume is 9.00 (6 zones). In the > 20% BMI, for the gradual group, the volume is 9.0 cc (6 zones), and for the rapid group is 10.0 cc (6 zones). Lastly, for the 61+ group in the < 20% BMI, in the gradual group, the volume injected is 10.0 cc (6 zones), and for the rapid cohort, the volume is 11.0 cc (6 zones). In the > 20% BMI, for gradual and rapid weight loss, the volume is 11 cc (6 zones).

When looking at the percentage difference for age increase, the mean percentage difference from 20–40 group to 41–60 group is 25.38% (SD: 8.82), and for 41–60 to 61+ group is 22.1025% (*p* = 0.68). When looking at the percentage difference from gradual weight loss groups to the rapid weight loss group, in Under < 20% BMI loss, the percentage difference between rapid and gradual is 16.84 (SD: 7.63), and in > 20% BMI loss is 14.91 (SD: 15.25) (*p* = 0.8544).

### 3.2 Females—Filler

In the ages 20–40 group, for < 20% BMI lost in a gradual and rapid manner, consensus was achieved to inject both zygoma (10/10) and pyriform (7/10). This not only addressed cheek projection but additionally lessened the formation of a tear‐trough and nasolabial fold by building up the bone platforms.

In the 20–40 group with > 20% BMI, in the gradual group, the malar region was additionally treated with pyriform and zygoma to help with midface volume loss. In the rapid weight loss group, temple (7/10), tear trough (7/10), malar region (7/10), lips (7/10), prejowl (7/10), piriform (9/10), and zygoma (9/10) were treated to combat global tissue descent and volume loss.

In the 41–60 age group, in < 20% BMI gradual and rapid weight loss groups, consensus was achieved in injecting temple, tear trough, zygoma, pyriform, naso‐labial fold, marionette lines, prejowl, and lips. Interestingly, in the > 20% groups, the same groups achieved consensus, but with the addition of jawline and subzygoma regions. This is due to the lack of jawline definition with massive weight loss and the hollowing seen in the subzygoma region.

In the 61+ group, for BMI < 20% consensus was achieved for temple (8/10), tear trough (7/10), zygoma (10/10), malar (8/10), pyriform (7/10), naso‐labial fold (7/10), marionette (10/10), jawline (7/10), prejowl (9/10), perioral (8/10), and lips (7/10). The rapid group was similar; however, it included subzygoma (7/10). In the > 20% BMI loss, gradual and rapid groups, consensus was achieved in all regions.

### 3.3 Males—Biostimulators

In terms of consensus, in the 20–40 age group, all categories of weight loss achieved consensus in treating Zones 2,3,4, and 5. In the 40–60 age category, for the < 20% BMI loss, a gradual consensus was achieved in treating the same zones: 2 (9/10), 3 (8/10), 4 (10/10), and 5 (8/10). In the rapid group, all zones achieved consensus. In the > 20% BMI gradual cohort, Zones 2–5 achieved consensus, while in the rapid group, all zones achieved consensus. In the over‐60 category, all zones in all weight loss categories achieved consensus.

To obtain total volumes, the mean cc of injection per zone that achieved consensus was added. The total volume for ages 20–40 BMI < 20% gradual and rapid is 5.0 cc (4 zones). In the same age group, for > 20% BMI reduction gradually the injection volume is 6.5 cc (4 zones), and for rapid is 7.0 cc (4 zones). In the 40‐ 60‐year‐old cohort, in the BMI < 20% reduction gradually, the volume injected is 6.5 cc (4 zones). In the rapid group, the volume is 8.5 (6 zones). In the > 20% BMI, for the gradual group, the volume is 8.5 cc (4 zones), and for the rapid group is 11.0 cc (6 zones). Lastly, for the 61+ group in the < 20% BMI, in the gradual group, the volume injected is 9.5 cc (5 zones), and for the rapid cohort, the volume is 10.5 cc (6 zones). In the > 20% BMI, for gradual weight loss, the volume is 11.0 cc (6 zones), and for rapid is 11.5 cc (6 zones).

When looking at the percentage difference for age increase, the mean percentage difference from 20–40 group to 41–60 group is 32.98% (SD: 12.20), and for 41–60 to 61+ group is 10.55% (SD: 0.86) (*p* = 0.0105), which is significant. When looking at the percentage difference from the gradual weight loss groups to the rapid weight loss group, in Under < 20% BMI loss, the percentage difference between rapid and gradual is 13.06 (SD: 12.92), and in > 20% BMI loss is 11.23 (SD: 11.27) (*p* = 0.8623).

### 3.4 Males—Filler

In terms of consensus, in the 20–40 age group with < 20% BMI lost in a gradual fashion, consensus was only achieved in the jawline group (7/10). In the rapid group, consensus was achieved in the malar region (8/10) and jawline (8/10) to provide lost cheek volume. In the > 20% BMI lost gradual group, consensus was achieved in the zygoma (7/10), malar (8/10), and jawline (8/10). In the rapid group, consensus was achieved in the same zones, but with the addition of the tear trough (8/10).

In the 40–60 age grouping in < 20% BMI loss, both the rapid and gradual groups achieved consensus in zygoma, malar, and jawline. In the > 20% lost gradually, consensus was achieved in the temple (8/10), tear trough (8/10), zygoma (8/10), malar (10/10), marionette (7/10), and jawline (9/10). In the rapid group, consensus was achieved in the same groups with the addition of subzygoma (7/10), pyriform (7/10), nasolabial fold (7/10), and prejowl (7/10).

In the age 61+ group, < 20% BMI loss in both gradual and rapid groups achieved consensus in tear trough, zygoma, malar, pyriform, marionette, and prejowl. In the > 20% BMI loss, the gradual and rapid groups achieved consensus in the temple, tear trough, zygoma, malar, pyriform, marionette, jawline, and prejowl. BMI > 20% in a rapid, temple, tear trough, zygoma, malar, pyriform, marionette, jawline, and prejowl.

## 4 Discussion

This consensus panel represents the first international effort to provide clinical guidance specifically for patients experiencing MDWL. The panel demonstrates the need to assess and address each treatment region individually in these patients. It also emphasizes the need to consider the appropriate range of volumization based on factors such as age, gender, and the degree of weight loss. While the consensus suggests initiating biostimulator treatment concurrently with weight loss to mitigate fat pad deflation and skin laxity, uncertainties remain regarding the optimal timing and dosing. Longitudinal studies are needed to confirm whether early intervention prevents the accelerated facial aging observed in MDWL patients.

Although the focus of this panel is on injectable rejuvenation, patients undergoing MDWL should also be counseled on the importance of resistance training, nutrition, as well as regular monitoring of laboratories [8]. Additionally, other diagnoses such as thyroid and other endocrine disruptions must be ruled out. MDWL patients are at an increased risk of developing sarcopenia, especially in some of the older patient populations [8]. In the prior consensus, panelists agreed that in the rapid weight loss population, the deflation of the superficial and deep fat pads of the face, the quality of the skin, as well as the sequelae of rapid weight loss, possibly affecting muscles, may all play a role in the appearance of accelerated facial aging. It has been previously demonstrated that in the massive weight loss population, their apparent age was 5.1 years older than their stated age [6]. It is therefore no surprise that the number of MDWL patients seeking facial rejuvenation will continue to rise in practices. This is especially true in the subset of patients who are very concerned about appearance, well‐versed in the anticipated changes, and are taking these medications to help lose stubborn weight. This contrasts with patients who are clinically obese and have decided to pursue medication in lieu of surgery, where they may present in a delayed fashion once the weight has been lost.

**FIGURE 6:**
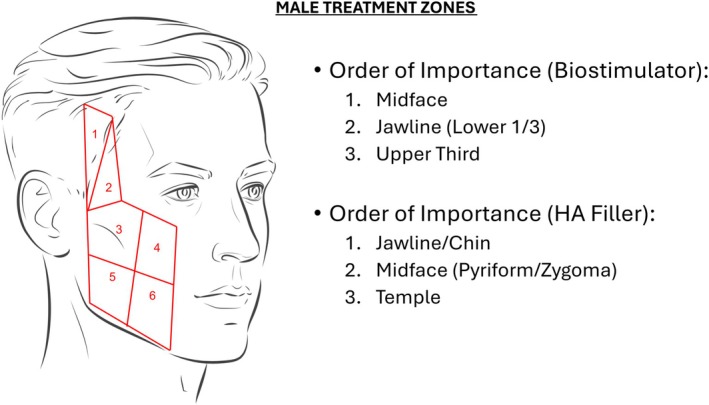
Goals of treatment for males.

**FIGURE 7:**
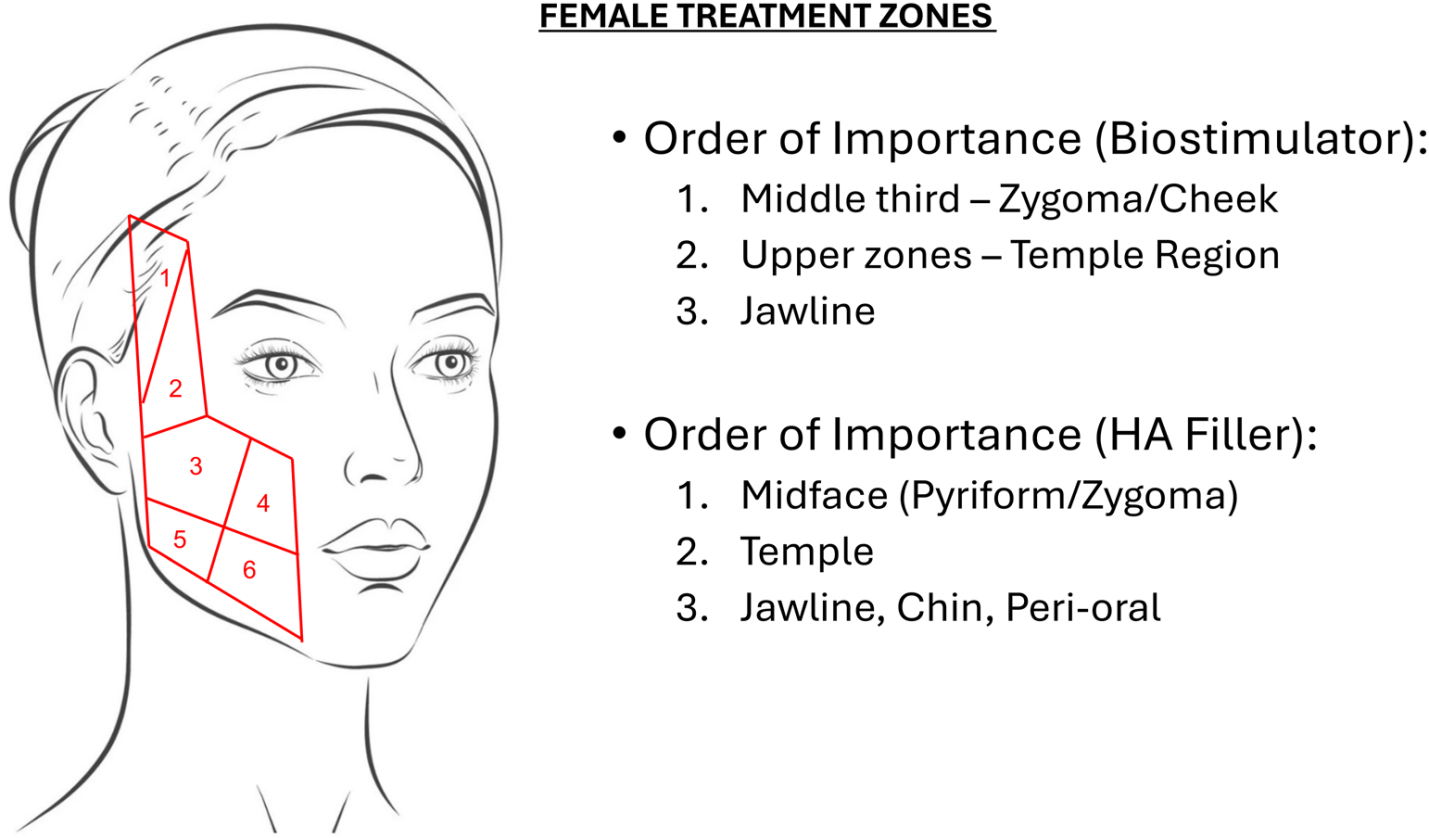
Goals of treatment for females.

When examining treatment areas, the number of anatomic regions as well as total treatment volumes increased with each ascending age group. The rapid weight loss group tended to also have larger treatment volumes and increased anatomic regions compared to the gradual weight loss group. As agreed upon in the prior consensus and seen in the literature, MDWL patients typically lose 5%–20% of their initial BMI, and bariatric patients lose ≥ 50% of their excess weight [8, 9]. Large amounts of weight loss can greatly impact the volumetric changes to the face, whereas gradual and smaller weight loss may allow the skin envelope to more effectively contract and accommodate, producing a less gaunt appearance. This is reflected by the consensus findings that certain groups, such as the older patients with poor skin quality, rapid weight loss, and a higher percentage of BMI loss, were all treated with larger volumes of biostimulator. Biostimulator, when compared to HA‐based injections, will carry less of a risk of overfilling if the patient were to regain weight or discontinue use of the GLP1 agonist. Biostimulators can also have a positive influence on adipogenesis and pre‐adipocytes, which greatly benefits these patients [10]. Given new technique development, injectors can be more precise with the volume distribution of biostimulators to individual treatment areas. Treatment goals can be seen in (Figures 6 and 7).

Interestingly, even with a relatively small sample size of panelists, the study revealed a statistically significant percentage increase in age‐related skin changes when comparing the 20‐ to 40 year‐old male group to the 41‐ to 60 year‐old male group. This suggests that the biological and structural changes in male skin become markedly more pronounced after the age of 40, despite individual variation, demanding the art of aesthetics. The findings support the idea that middle age is a critical period for the onset of visible skin aging in men, with measurable shifts in skin characteristics such as thickness, elasticity, and texture. Male skin differs significantly from female skin due to various physiological factors, one of the most notable being increased skin thickness [11]. On average, male skin is about 20%–25% thicker than female skin in early adulthood [11, 12]. This greater initial thickness, however, does not mean that male skin ages more slowly in all respects. In fact, a landmark study by Shuster et al. in 1975 was among the first to demonstrate that male skin thickness declines steadily and linearly with age, beginning as early as 20 years old [11]. This means that while men start with a structural advantage, their skin undergoes a consistent degradation in thickness over time, which can eventually lead to sagging, deep wrinkles, and reduced barrier function. In contrast, female skin maintains relatively consistent thickness until around age 50, at which point it begins to thin more rapidly, particularly after menopause [11].

Skin thickness may play a larger role in how the skin contracts and accommodates the volume changes in the MDWL patient. This is an important consideration when considering treatment volumes for the respective regions. The age‐related changes in the elastic properties and thickness of human facial skin were measured by Takema et al. [13] They determined that the quantitative and qualitative changes in elastic fibers in facial skin can have a considerable effect on the thickness and physical properties of the skin and its ability to contract [13]. Clinically, this suggests that baseline skin assessment is essential, and in some cases, delaying volumization until postweight‐loss stability may avoid overtreatment.

After consensus discussion, the ideal scenario is to firstly time injection with biostimulator to compensate for weight loss and skin changes, but also to compensate for larger volume loss in older patients or with significant weight loss. Previously, it was described that weight loss patients sustain volume changes along the midfacial region, periorbital region, and temples, as well as develop an increased number of facial wrinkles [14]. Given these findings, it is no surprise that temple filling was added as weight loss increased or with aging. This is because temple injections can help with restoring atrophic temples, but also by utilizing it as a cantilever effect on the midface. It was shown in a randomized controlled trial that poly‐l‐lactic acid injected into the extended temple had a greater lifting effect on the face and may serve as an adjunct to traditional temple injections [7]. It is important, however, to understand the proper dilution and injection technique of poly‐l‐lactic acid. When diluted using 8 mL of sterile water and 1 cc of lidocaine without epinephrine, it is crucial to ensure that the injection is performed in the appropriate subcutaneous plane. Consensus was achieved in that the use of a cannula is preferred, with the tip of the cannula always able to be palpated during injection. This will preferentially place the product into the proper superficial plane.

In terms of the utilization of hyaluronic acid fillers, as noted by the increased number of anatomic regions that obtained consensus, they really play a role in the volumization of the MDWL patient. While biostimulators can help provide luster and an increase in collagen to the skin, HA filler can be preferentially placed in areas devoid of volume or to help with midface projection, which is a common sequel of MDWL. One technique preferred among the panelists is placing filler deep on the pyriform aperture and anterior malar region to help correct some finer tear troughs or nasolabial folds produced by a lack of midface support. In addition to changes caused by weight loss, it is well known that the face ages predictably in a clockwise fashion with clear midface retrusion as the face ages [15, 16]. This senile midface retrusion, coupled with the rapidity of weight loss, can be partially mitigated with strategically placed filler.

As always, regardless of volumes utilized, it is of utmost importance to respect safe injection technique, as well as reconstitution of the product. Although not covered by the panel, product selection is equally paramount. As a rule, products placed on bone or supraperiosteal should be of higher G' and cohesivity mimicking the effects of bone. This property is also advantageous for a targeted treatment to provide lift and projection, particularly in the midface and cheek, where the skin tends to be thicker and more resistant to expansion.

One important part of the multidisciplinary approach often forgotten by clinicians is the need for proper skin care and utilization of products to boost hydration, such as skin boosters. As the face ages, especially in the postmenopausal patient, the need for hydration and moisture is paramount. Further adding fuel to the fire, the use of GLP1 agonists can suppress water intake, and anecdotal reports by patients point toward a lack of skin hydration18. By adding in some topical treatments or superficially placed HA bolus, the youthful luster can be restored.

## 5 Limitations

This study is not without limitations. First, given the relatively smaller size of the consensus, clinicians should use these recommendations to help guide treatment rather than adhere very strictly to them. Second, the concept of facial changes with MDWL is a rather new concept. As medications continue to be used and modified, there may be further guidelines or recommendations that would be appropriate. Lastly, this panel focuses primarily on nonsurgical rejuvenation, which, in certain patients, cannot replace the need for surgical intervention.

## 6 Conclusion

In conclusion, this consensus panel represents the first international effort to provide clinical guidance specifically for patients experiencing MDWL. When creating a treatment plan for the MDWL patient, following them throughout the weight loss journey is essential to understand the more global volume changes that they may be observing. This is dynamic and is by no means a one‐size‐fits‐all approach. With increasing age and rate of weight loss, larger volumes of biostimulators and fillers may be needed to restore the face to a more youthful appearance.

## Author Contributions

A.N.: panelist, study design, manuscript write‐up. M.T.S., S.D., H.C., S.F., L.A., J.F., K.F., A.H., M.A.A., J.F.: panelist, study design, manuscript edits. I.P.: study design, manuscript edits. T.S.: study design, manuscript write‐up, revisions, analysis.

## Ethics Statement

All images used are generated avatars. No informed consent required.

## Conflicts of Interest

Andreas Nikolis is or has been a consultant, investigator, trainer, and speaker for Galderma (Lausanne, Switzerland), Allergan (Dublin, Ireland), Prollenium (Ontario, Canada), and Merz (Frankfurt, Germany). Sabrina G. Fabi and Alessandra Haddad are or have been speakers, investigators, trainers, and consultants for Galderma. Luiz Avelar is or has been a speaker, investigator, and consultant for Galderma. Michael Somenek is or has been a speaker, investigator, trainer, and consultant for Galderma. Hugues Cartier is or has been an investigator and consultant for Galderma. Steven Dayan is or has been a consultant, researcher, and speaker for Galderma. Inna Prygova is an employee of Galderma. Jeff Huang has no conflicts of interest to report.

## Data Availability

The data that support the findings of this study are available from the corresponding author upon reasonable request.
