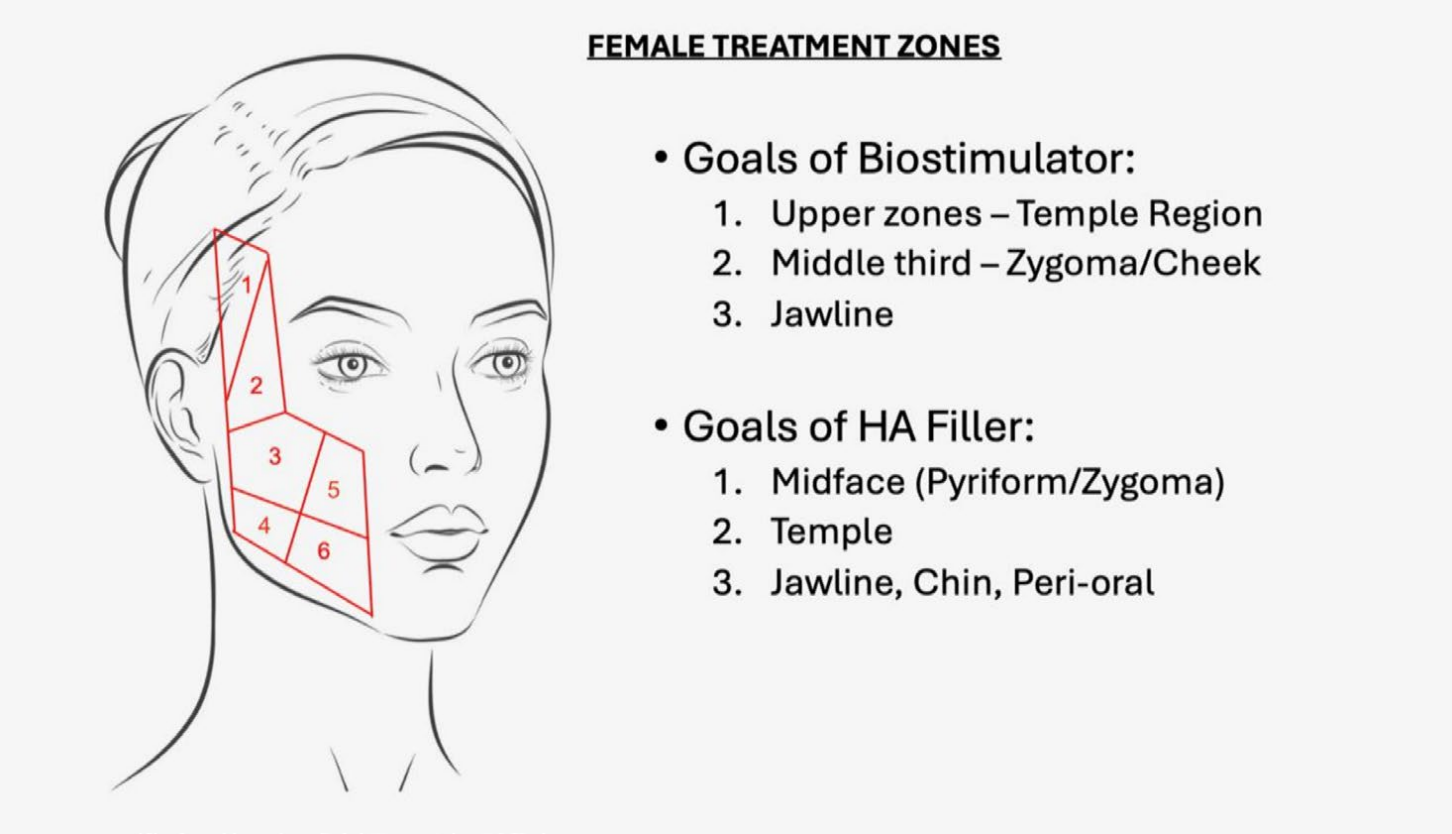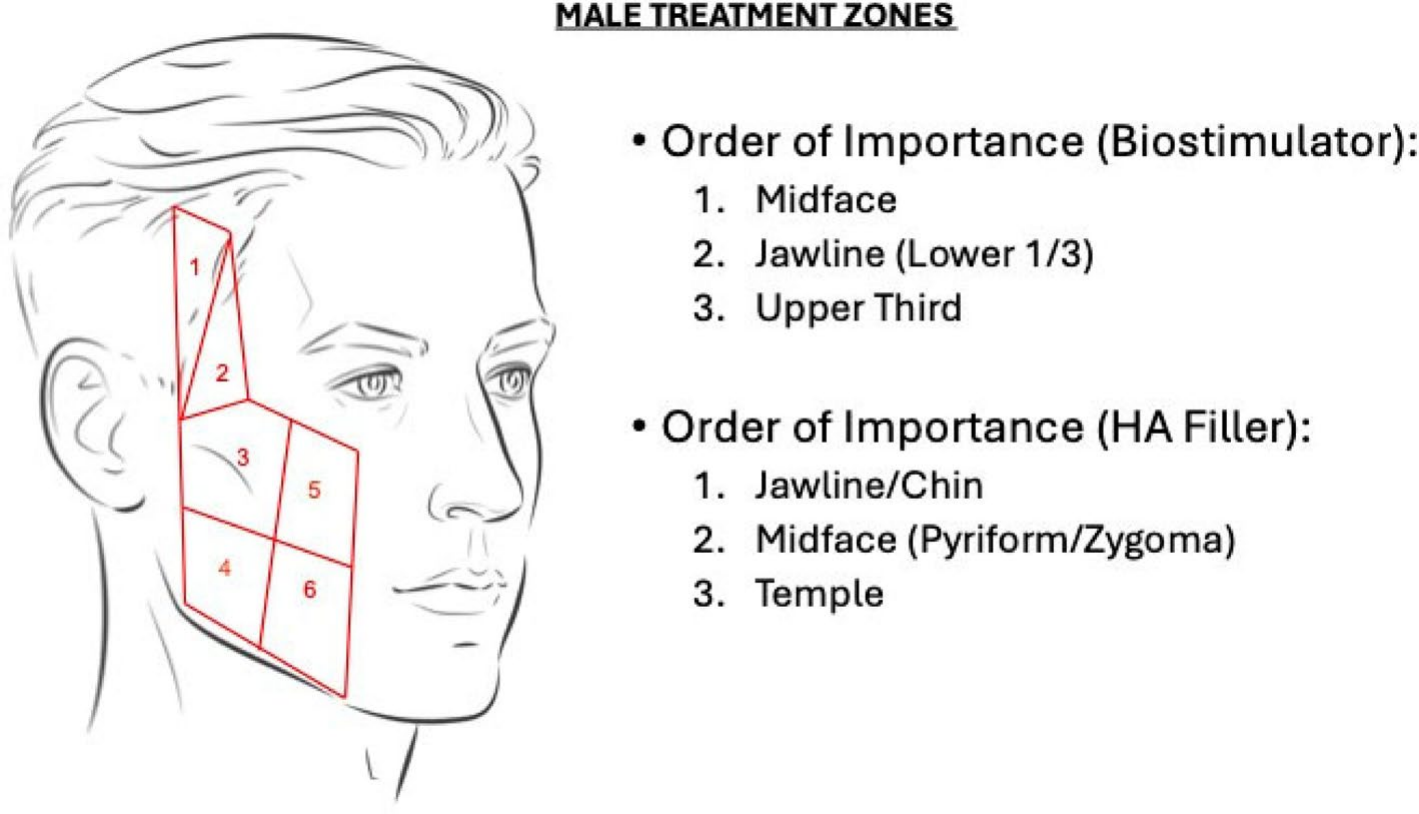# Correction to “Managing Aesthetic Needs in Prescription Medication‐Driven Rapid Weight Loss Patients: Results of an International Consensus. The Clinician Perspective”

**DOI:** 10.1111/jocd.70781

**Published:** 2026-03-08

A. Nikolis, M. T. Somenek, S. Dayan, H. Cartier, S. G. Fabi, L. Avelar, J. Franco, K. Frank, A. Haddad, M. A. Alsufyani, J. Huang, I. Prygova, and T. Safran, “Managing Aesthetic Needs in Prescription Medication‐Driven Rapid Weight Loss Patients: Results of an International Consensus. The Clinician Perspective,” *Journal of Cosmetic Dermatology* 25, no. 1 (2026): e70644, https://doi.org/10.1111/jocd.70644.

In Figures 6 and 7, the numbers 4 and 5 on the diagram are inadvertently swapped. These labels should be congruent across all figures.

We apologize for this error.